# Role of Oxidative Stress in Retinal Disease and the Early Intervention Strategies: A Review

**DOI:** 10.1155/2022/7836828

**Published:** 2022-10-14

**Authors:** Jun Wang, Mengling Li, Ziyue Geng, Saadullah Khattak, Xinying Ji, Dongdong Wu, Yalong Dang

**Affiliations:** ^1^School of Basic Medical Sciences, Henan University, Kaifeng, China; ^2^Henan International Joint Laboratory for Nuclear Protein Regulation, Henan University, Kaifeng, China; ^3^College of Acu-Moxibustion and Massage, Shaanxi University of Chinese Medicine, Xianyang, China; ^4^School of Clinical Medicine, Henan University, Kaifeng, Henan, China; ^5^Sanmenxia Central Hospital, Sanmenxia, Henan, China

## Abstract

The retina, owing to its cellular anatomy and physical location, is susceptible to generating reactive oxygen species (ROS), which are associated with several major retinal diseases. When ROS exceeds the body's natural antioxidants, the retina is in a state of oxidative stress, which is recognized as the pathogenesis of retinal diseases. The early stage of the pathogenic process is an adaptive change in which oxidative stress and endogenous defense mechanisms occur. If no treatment is applied, the retinal diseases will progress to the pathological stage with neuronal and vascular dysfunction or damage and even blindness. This review summarizes the role of oxidative stress in several common retinal diseases, including retinitis pigmentosa, age-related macular degeneration, diabetic retinopathy, glaucoma, and retinopathy of prematurity. In addition, we discuss the early intervention strategies for these diseases. An outline is provided to identify potential intervention targets for further research. Early intervention for retinal diseases is necessary and urgent and may offer hope to improve patients' quality of life through functional vision.

## 1. Introduction

The retina is an extension of the brain and is a highly oxygen-consuming organ in the body, relying more on aerobic glycolysis than the brain; it is also highly sensitive to various stimuli [1]. Furthermore, the retina is extremely metabolically active and rich in polyunsaturated fatty acids, which are vulnerable to lipid peroxidation [2]. Under normal conditions, reactive oxygen species (ROS) in the retina are related to physiological signaling and protective mechanisms via the prosurvival extracellular signal-related kinase 1/2 pathway and endoplasmic reticulum stress signaling. ROS production-induced oxidative stress may contribute to the pathogenesis of several retinal degenerative diseases, including diabetic retinopathy (DR), retinal vascular occlusion, retinitis pigmentosa (RP), age-related macular degeneration (AMD), glaucoma, and retinopathy of prematurity (ROP).

The number of people with retinal diseases, including AMD, DR, and glaucoma, is expected to more than double by 2050 [3]. This prediction indicates that there will be a healthcare crisis that not only affects patients with visual disturbance but also the caregivers and entire healthcare system. Thus, there is an urgent need to focus on the prevention of and protection against retinal diseases. This review includes the necessary recommendations and neuroprotective strategies for preventing the progression of vision loss and blindness in individuals with retinal diseases.

In living cells, oxidation-reduction reactions may generate metabolic byproducts—free radicals, which are ROS and reactive nitrogen species (RNS) [4]. Free radicals are a class of molecules containing one or more unpaired electrons. ROS can be generated via multiple pathways, including endogenous and exogenous substances [4]. ROS are highly active and may react with biomolecules, e.g., proteins, lipid membranes, and DNA, leading to cell damage or functional impairment. Oxidative stress is a condition of oxidation-reduction imbalance characterized by elevated levels of ROS and free radicals or a reduction of antioxidants [5, 6]. Under physiological conditions, the generation of ROS and the scavenging of free radicals may reach a dynamic redox balance. When various excess stimuli (endogenous and/or exogenous) occur, ROS accumulate massively. This may lead to oxidative stress in the corresponding cells and organs [7]. Continuous oxidative stress may ultimately damage cells in target organs. Therefore, the inhibition of ROS generation and scavenging of excessive ROS by various pathways have been used as therapeutic strategies for the treatment of eye diseases. In this review, we will introduce the mechanisms of ROS generation in several retinal diseases, summarize the pathogenetic roles of oxidative stress in their development, and discuss the early intervention pathway of antioxidative stress.

## 2. Physiological Role of Free Radicals and ROS in Organisms

The free radicals in the human body can be divided into oxygen and nonoxygen radicals. Free oxygen radicals are the predominant category derived from molecular oxygen, and their proportion of oxygen radicals is approximately 95% of the total radicals. Oxygen radicals include hydrogen peroxide (H_2_O_2_), hydroxyl radicals, superoxide anions (O2^−^·), nitroxides, and peroxynitrite, which are defined as ROS and RNS. ROS/RNS can be produced by the metabolism of normal cells and exogenous stimuli, e.g., chemical drugs, high-pressure oxygen, radiation, mental stress, insomnia, smoking, and pollution [8] Free radicals are highly reactive and short-lived intermediates that have one or more unpaired electrons and exist independently on their own [9, 10]. Under normal conditions, free radicals are important for maintaining homeostasis and defending the body against hazardous invasion [11] by (1) enhancing phagocytosis of leukocytes, (2) promoting synthesis of prostaglandin and lipoxygenases, and (3) relaxing vascular smooth muscle and modulating blood pressure. Endogenous ROS are derived from the mitochondria by escaping electrons to molecular oxygen [12]. The generation sources include NADPH oxidases (Nox), xanthine oxidase, and lipoxygenase on the membranes of endothelial cells and phagocytes [2]. In most cases, the terms “free radicals” and “ROS” are regarded as interchangeable [12]. Healthy organisms always generate low levels of free radicals, and antioxidant defense systems may scavenge them rapidly before they cause oxidative damage to the cell. If the dynamic redox balance is imperfect, ROS/RNS-mediated damage may occur continuously. In other words, oxidative stress injury is generated when the speed of free radical production exceeds the capacity of the cellular defense system, for example, exposure to high oxygen pressure or ionizing radiation [8]. Oxidative stress damages biological macromolecules, e.g., nucleic acids, proteins, and lipids by peroxidation, degeneration, crosslinking, and breakage. Finally, oxidative stress can cause injuries to cell structures and functions, as well as to the tissues and organs in the body [11]. Thus far, increasingly more diseases/disorders are recognized gradually by linking them directly or indirectly with oxidative stress [8]. Some of these disorders are due to free radicals, whereas others may be only secondarily involved. Tissue injured by various processes, such as trauma, toxic substances, and infections, may undergo free radical damage more rapidly than healthy tissues. Tissue destruction and degeneration may increase oxidative damage through processes including metal ion release, phagocyte activation, and disruption of mitochondrial electron transport chains. Iron chelation, superoxide dismutase, catalase, vitamins (C and E), and antioxidants (flavonoids) could have protective effects under various experimental conditions, which further indicate the key role of free radicals in many disorders [13–16]. Additionally, an increasing number of small-molecule drugs have been developed and implemented to target ROS [17].

## 3. Oxidative Stress and Retinal Pathology

Oxidative stress plays a prominent role in the pathogenesis of many degenerative retinal diseases, such as AMD, DR, and RP. Normal, healthy retinal cells are susceptible to significant light exposure, which may lead to the generation of abundant ROS [18]. However, under pathological conditions, the normal homeostatic mechanisms are destroyed. When the relationship of prooxidative stress and antioxidative stress signaling is unbalanced, it may lead to excessive oxidative stress, inflammatory responses, blood-retinal barrier injury, and retinal tissue damage [18, 19]. Retinal diseases, including photoreceptor degeneration, diabetic retinopathy, and retinal ganglion cell injury, always involve the same process of oxidative stress and apoptosis in the final pathological stages. This review will provide a summary of the effects of oxidative stress in several congenital retinal diseases and possible early intervention strategies.

### 3.1. Oxidative Stress in RP

**Figure 1:**
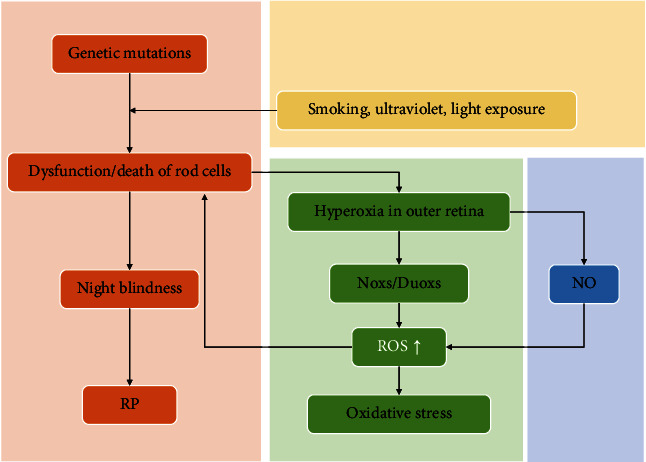
Oxidative stress is involved in the rod cell death-induced clinical feature of RP. ROS: reactive oxygen species; RP: retinitis pigmentosa.

RP is an inherited retinal disease caused by different genetic mutations. The pathological process leads to photoreceptor cell degeneration (successive rod and cone cell loss) and eventually results in retinal pigmented epithelium (RPE) dysfunction [2]. The prevalence rate is 1 in 4000 persons worldwide, and there are >1.5 million patients with RP worldwide [20]. The early phenotype of RP is difficult to observe at night, and loss of peripheral vision is caused by apoptosis of rod photoreceptors. Then, vision loss becomes more restricted to the central visual field and is eventually lost [20]. Studies have demonstrated that progressive visual loss in RP is associated with the loss of photoreceptor cells and oxidative stress (Figure 1) [21, 22]. Clinical evidence also showed that 8-oxo-7,8-dyhydro-2′-deoxyguanosine (8-oxo-dG) and protein carbonyl contents were increased in the vitreous and aqueous humor of patients with RP [23, 24]. This finding indicates that oxidative stress exists in the ocular tissues of patients with RP. In the outer nuclear layer, rods account for 95% of the cells and are more metabolically active than cones. These cells gradually decrease in number as RP progressed [24]. Subsequently, retinal oxygen demand and usage also decrease. Retinal circulation is not regulated; therefore, the oxygen supply to the outer retina increases spontaneously [24]. Hyperoxia in the outer retina can induce Nox and dual oxidase, but not xanthine oxidase, to produce excessive amounts of ROS and contribute to tissue damage [25]. Hyperoxia in the outer retina induced by rod degeneration may activate Nox enzymes and may also activate the high levels of Nox2 in microglia, which have been demonstrated to migrate into the outer retina in RP. This leads to an increase of superoxide radicals and oxidative damage [25, 26]. High levels of nitric oxide in the outer retina are another indirect oxidative stress factor that may react with superoxide radicals to form highly reactive and damaging peroxynitrite radicals. Hence, inhibition of Nox and nitric oxide synthase (NOS) together with antioxidative stress by scavenging ROS directly should be regarded as a strategy to help maintain photoreceptor function in patients with RP.

#### 3.1.1. RP: Intervention Strategy at an Early Stage

RP is a family of inherited diseases. Over 250 gene mutations are associated with the cause of rod death, and it is likely the major pathogenesis of RP [27, 28]. Therefore, RP is difficult to cure at the genetic level despite the development of genotype-phenotype technology that may already characterize each causal gene in RP [29]. Currently, there is no specific pharmacological or genetic treatment for RP [27]. Antioxidants have shown promise as an intervention to delay the reduction of cone cell loss and retard the progress of RP [30]. However, the clinical evidence has not been clarified. The application of antioxidant supplements, such as vitamins A and E, beta-carotene, omega-3, docosahexaenoic acid (DHA), zinc, and docosahexaenoic acid, might be able to slow the decline in peripheral field function [20, 30]. The oral antioxidant N-acetyl cysteine (NAC) for RP was studied in a phase 1 clinical trial [31]. However, its efficacy should be addressed in future studies. In addition, a study showed that smoking is detrimental to patients with RP, as it worsens macular function and structural integrity [32]. Hence, smoking may not benefit patients with RP. Furthermore, ultraviolet and blue light exposure may accelerate vision loss in RP [33, 34]. Wearing protective goggles is a simple and effective method to prevent light damage. Regular to moderate physical activity, not endurance exercise without adaptable physical training, may protect against ROS/RNS damage in eye diseases, including RP [35]. Intraocular pressure may affect the circulation of the outer retinal layer. It is possible that an obvious reduction in choroidal blood flow through the elevation of intraocular pressure may prevent sufficient oxygen supply from the choroid [36]. Therefore, it is reasonable to assume that appropriate eyeball massage may be a preconditioning intervention to the retinal outer layer to improve endogenous antioxidative stress during the early stage of RP. Finally, prevention of consanguinity and reinforcement of genetic counseling before birth is very important in reducing the risk of RP. Antioxidant gene modification may be a potential treatment for late stages of RP in the future; although, transgenic overexpression is not applicable to humans.

### 3.2. Oxidative Stress in AMD

**Figure 2:**
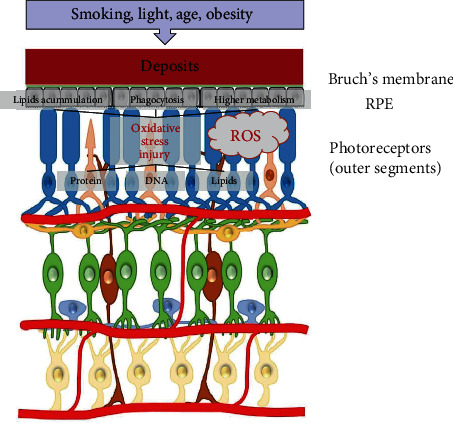
Oxidative stress in AMD. ROS are increased in RPE and photoreceptors since the exposure of environmental risk factors in the early stage of AMD. ROS lead to the deposits of lipid substances, phagocytosis disability, and cell injury in protein, lipids, and DNA, until cell death. AMD: age-related macular degeneration; ROS: reactive oxygen species; RPE: retinal pigmented epithelium.

AMD is a chronic, irreversible disease that primarily affects central vision and is an important cause of blindness worldwide. Accumulations of lipid and protein between the RPE and Bruch's membrane (BrM); loss of photoreceptors, RPE, and retinal neurons; and neovascularization are the main pathological processes. Multiple risk factors have been reported for AMD including age, light exposure, smoking, obesity, hypertension, poor antioxidant intake, and a hereditary component [37, 38]. Many studies have demonstrated that oxidative stress is involved in the pathogenesis of AMD [39–42]. The levels of oxidative stress markers, including malondialdehyde, protein carbonyls, and 8-hydroxy-2-deoxyg, are increased significantly in the blood serum of patients with AMD [37, 43]. This finding indicates that systemic oxidative stress is associated with AMD. Additionally, a higher level of carboxyethylpyrrole (CEP) and damaged proteins was detected in the BrM of donor eyes with AMD [44]. CEP is formed from DHA during oxidative stress. The outer segments of photoreceptors are abundantly composed of DHA, which increases their vulnerability to oxidative damage. Under stress conditions, photoreceptors must metabolize continuously to regenerate outer segments, producing a unique source of ROS in RPE cells [45]. RPE cells are responsible for phagocytosis of photoreceptor outer segments. ROS overaccumulation may cause disorders in cell structure and function, which in turn increases ROS generation. Taken together, high oxygen metabolism, continuous light exposure, and high concentrations of polyunsaturated fatty acids make the retina prone to retinal damage. Thus, oxidative stress plays important roles in the pathophysiology of AMD (Figure 2).

#### 3.2.1. AMD: Intervention Strategy at an Early Stage

(1) Cigarette smoking has been shown experimentally to be directly linked to the development of AMD and is a major risk factor in epidemiological studies [46, 47]. Therefore, quitting smoking as early as possible is a good strategy to prevent AMD. (2) One of the mechanisms of retinal injury in AMD is the interaction between light exposure and photosensitive molecules (rhodopsin and lipofuscin) [48, 49]. Excessive activation of rhodopsin and light conduction can cause the formation of ROS from the DHA content of the outer segment membranes of the rod and induce photoreceptor cell degeneration [49]. It has been suggested that shorter wavelengths have a higher risk of retinal injury than longer wavelengths [50]. Avoiding exposure to blue light may prevent the increased formation of ROS by photosensitive molecules [51]. (3) Experimental studies have shown that an antioxidation-deficient diet is associated with lipofuscin accumulation and photoreceptor degeneration in the RPE [52]. Increased antioxidants in the diet (vitamins A, C, and E and carotenoids) or serum could protect against AMD progression [49]. A longitudinal clinical study also indicated that the consumption of antioxidants/zinc could decrease the risk of early AMD in a highly susceptible group [53]. Moreover, the topical antioxidant OT-551 (0.45%), investigated in a single-center phase II trial, may improve best-corrected visual acuity [54]. Age-related eye disease studies, antivascular endothelial growth factor (VEGF) injections, and laser therapy are also useful for controlling the progression of wet AMD [55, 56]

### 3.3. Oxidative Stress in DR

**Figure 3:**
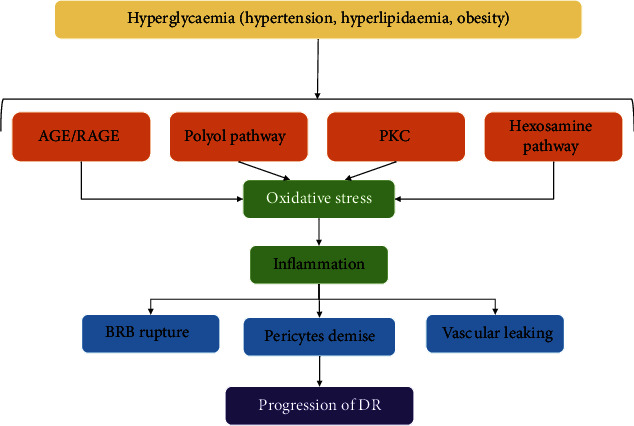
The driving mechanism of oxidative stress in the progression of DR. PKC: protein kinase C; AGEs: advanced glycation end-products; BRB: blood-retinal barrier; DR: diabetic retinopathy.

DR, a progressive microvasculature complication of diabetes, is one of the most common causes of blindness in adults of working ages [57, 58]. Approximately 90% of diabetic patients develop DR complications within 25 years of diagnosis [59]. Oxidative stress and inflammation are considered to play key roles in the pathogenesis of DR because the retina has high vascularization and long-term light exposure [60]. Chronic hyperglycemia exposure, resulting in increased ROS production, makes microvessels more vulnerable to oxidative stress. The disturbance of redox homeostasis contributes to the death of neurons in the retina, followed by the rupture of the blood-retinal barrier and increased vascular permeability, leading to advanced DR. In retinal cells under oxidative stress conditions, excessive ROS directly acts on protein and DNA or indirectly acts as a second messenger to affect the pathogenesis of DR (Figure 3) [58, 61, 62]. As the main source of intracellular ROS, mitochondria are abundant in photoreceptors, which are the major O2- contributor in DR [63]. Studies have shown that mitochondrial dysfunction in turn affects the production of ROS in retinal cells, activity of optic nerve cells, and function of photoreceptors. ROS accumulation causes further deterioration [64]. Besides, mitochondrial dysfunction may reduce mitochondrial energy production, leading to optic nerve degeneration [65, 66]. Additionally, the accumulation of byproducts caused by metabolic abnormalities induced by hyperglycemia, e.g., the activation of protein kinase C, hexosamine, polyol flux, and advanced glycation end-products (AGEs), induces oxidative stress through ROS/RNS formation, leading to the death of retinal neurons [58, 67]. Oxygen-derived free radicals, such as hydroperoxyl species, have been shown to cause lipid peroxidation, contributing to the production of ROS to facilitate the senescence of RPE cells, leading to the progression of DR [68–70]. Therefore, oxidative stress plays an important role in DR progression.

#### 3.3.1. DR: Intervention Strategy at an Early Stage

Current therapies for DR, such as anti-VEGF treatment, laser therapy, vitrectomy, and glucocorticoids, focus on the late stage, which may reduce visual loss by temporarily protecting retinal vessels [71, 72]. However, it is still difficult for patients with severe vision loss to reestablish normal vision. As a major factor in the progression of DR, oxidative stress is a hot target at an early stage. Nox, the main enzymatic source of ROS, has been demonstrated to be a direct risk factor for DR, and inhibitors of the Nox family have been studied to prevent the development of DR. For example, diphenyleneiodonium (a Nox inhibitor) can suppress ROS generation, alleviate blood-retinal barrier breakdown, and recover the death of retinal capillary endothelial cells [73, 74]. Nox 1/4 specific inhibitors, GKT136901 and GKT137831, have shown potent effects in DR treatment [75]. Polyphenols are antioxidants that are abundant in vegetables, fruits, beverages, whole grains, etc. Studies have demonstrated the protective effects of DR against various polyphenols, including green tea polyphenols, chlorogenic acid, curcumin, beta-glucogallin, and cocoa polyphenols [76–78]. Resveratrol, the most studied polyphenol, has been shown to activate antioxidant enzymes and inactivate NOS activity with a decrease in ROS/RNS in the blood and retina in various experimental models [79–81]. The ROS scavengers NAC and duloxetine are effective in the early stages of DR [82]. Nuclear factor erythroid 2-related factor 2 (NRF2) binds to antioxidant response elements to regulate antioxidant protein levels and fight against oxidative stress. Recent studies have shown that activation of the NRF2 pathway provides new insights for DR treatment. The novel NRF2 activator dh404 provides potential protection to the retina in diabetes, including vision-threatening breakdown of the blood–retinal barrier [83, 84]. Herein, the inhibition or clearance of ROS generation provides an early candidate strategy for blindness caused by DR.

### 3.4. Oxidative Stress in Glaucoma

**Figure 4:**
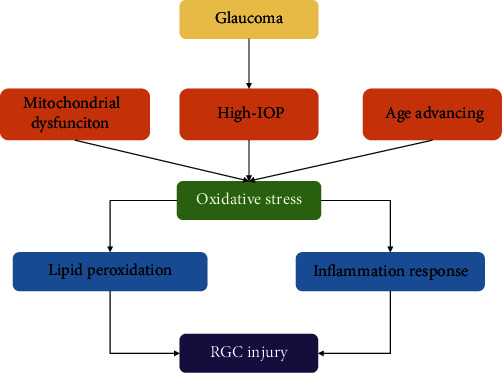
The role of oxidative stress in glaucoma RCG injury. IOP: intraocular pressure; RGC: retinal ganglion cell.

Glaucoma is a progressive optic neuropathic disease characterized by retinal ganglion cell (RGCs) degeneration and synaptic loss of dendrites and axon terminals and is the most common cause of irreversible blindness worldwide [85]. It was estimated that the number of people with glaucoma will reach 111.8 million by 2040 globally because of the increase in the number of people of advanced age [86, 87]. Although intraocular pressure is the only known risk factor for glaucoma, increasing studies have shown that oxidative stress plays an important role in the pathogenesis of glaucoma [88, 89]. Increased intraocular pressure and advancing age are closely implicated in the pathogenesis of glaucoma. Oxidative stress occurs in the eyes and may explain the potential mechanism underlying the development of glaucoma [90–92]. Moreover, mitochondrial dysfunction is largely related to oxidative stress in the pathogenesis of glaucoma [93, 94]. Especially in the aging retina, oxidative stress and lipid peroxidation are the major risks to activate the inflammatory response, leading to RGC death and apoptosis, aggravating glaucoma (Figure 4) [95]. Exposure to sunlight and a high-oxygen environment causes higher oxidative stress in the eyes than in other tissues, which can further damage the eye tissue [96, 97]. The trabecular meshwork (TM) is the most sensitive tissue in the eye and is vulnerable to oxidative stress [98]. Studies have shown that increased 8-OH-dG levels are found in the TM of patients with glaucoma, indicating the occurrence of DNA oxidative stress damage [99, 100]. In addition, the accumulation of ROS, particularly H_2_O_2_, was also detected in TM cells, which reduced antioxidant activity and inhibited the secretion of adhesion molecules to TM cells, contributing to cytoskeleton reorganization and eventually resulting in cell loss [91, 101, 102].

#### 3.4.1. Glaucoma: Intervention Strategy at an Early Stage

Early diagnosis and intervention are important to prevent visual loss in patients with glaucoma due to irreversible blindness [103]. Based on the increasing evidence of oxidative stress in glaucomatous tissues, the levels of biomarker candidates related to oxidative stress, e.g., protein carbonyls and AGEs, have been shown to increase significantly in the blood and aqueous humor samples of patients with glaucoma [104]. Oxidative stress is an important risk factor for ocular hypertension, which is a target of glaucoma treatment [105, 106]. Drugs with antioxidant properties, such as valproic acid and spermidine, have been reported to prevent glaucomatous retinal degeneration in glaucoma mouse models [107, 108]. The grafting of antioxidant molecules to drug carriers can also effectively reduce the intraocular pressure in glaucoma [109]. The activation and recruitment of microglia and astrocytes to the edge of the optic nerve are early characteristics of glaucoma [110–112]. Inhibition of ROS can inhibit the byproducts of electron leakage along the electron transport chain during cell respiration and improve mitochondrial dysfunction, contributing to the delay in glaucoma progression [95, 113, 114]. Additionally, data based on a large population have shown that quitting smoking and moderate-intensity aerobic exercise may reduce the risk of glaucoma [115–117]. A balanced diet, including vegetables, omega fatty acids, and coffee, may help prevent the occurrence or progression of glaucoma [118]. Studies showed that oxidative stress might be a potential target for the prevention and treatment of glaucoma in the early stages; although, there is no direct clinical evidence.

### 3.5. Oxidative Stress in ROP

**Figure 5:**
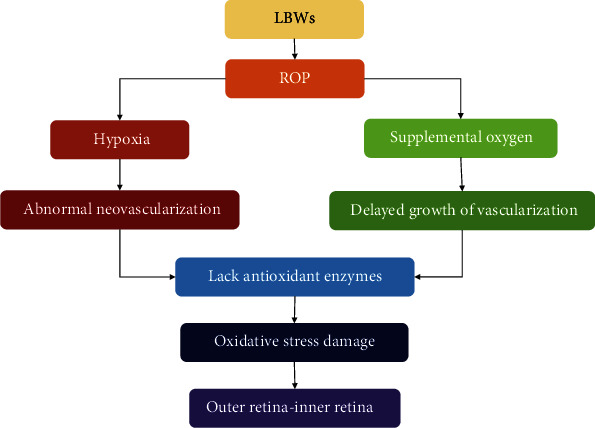
The increased ROS level plays a key role in the pathogenesis of ROP. LBWs: low-birth-weight infants; ROP: retinopathy of prematurity.

ROP is a complex eye disease characterized by retinal neovascularization in low-birth-weight preterm infants (LBWs), and it is the most common cause of blindness in children [119, 120]. Oxidative stress is a physiological redox imbalance caused by excessive ROS/RNS. It plays a key role in the pathogenesis of ROP and significantly increases the mortality and morbidity of ROP in very LBWs (Figure 5) [119, 121, 122]. There are two phases of ROP: (1) abnormal retinal neovascularization motivated by hypoxia and (2) delayed growth of retinal vascularization induced by supplemental oxygen [123–125]. Clinical data showed a connection between the incidence of ROP in LBWs and unrestricted oxygen exposure, and the corresponding findings have shown that reduced oxygen saturation can decrease the incidence of ROP [126]. For newborns, oxidative stress is a challenge during the process of birth itself. The sharp postnatal transition from a lower oxygen content with an intrauterine environment into a higher oxygen content environment causes oxidative stress in infants, and most preterm infants lack antioxidant enzymes and chemical antioxidants because the increase in key antioxidant enzymes such as superoxide dismutase only occurs in late pregnancy, showing a lower antioxidant ability in premature infants [127, 128]. Retinal hyperoxia in LBWs due to the lack of autoregulation of the blood network in the retina causes an imbalance of prooxidants and antioxidants, contributing to the inflammation of retinal tissue, eventually resulting in the development of ROP [129–131]. Moreover, in ROP, damage of the outer retina occurs along with the increased ROS levels in the inner retina [131, 132].

#### 3.5.1. ROP: Intervention Strategy at an Early Stage

Early intervention is an efficient strategy to control the incidence of ROP, a preventable ocular disease [133]. According to the early treatment of ROP, cryotherapy and laser therapy are now effective methods to prevent ROP in preterm infants [134–136]. Strict oxygen limitation effectively reduces the incidence of ROP in LBWs. Flavonoids are a group of antioxidants present in the diet [137, 138]. For example, green tee has been demonstrated to prevent ROP through the inhibition of corneal neovascularization in animal models [139, 140]. Bilberries, a natural source of anthocyanins with high antioxidative properties, can significantly render lipid peroxidation and neovascular proliferation and protect the retinal vasculature after high oxygen therapy, contributing to the suppression of ROP progression [140, 141]. Other flavonoids, e.g., baicalin and luteolin, have also been shown to protect against ROP because of their ability to scavenge ROS [142, 143]. Additionally, prenatal supplementation with antioxidants, such as carotenoids and vitamins (A and E), helps to promote normal fetal growth, resulting in a reduction in the birth rate of preterm infants; although, there is no clinical evidence that it can prevent ROP in LBWs [144–147]. Moreover, supplementation with essential fatty acids, e.g., *ω*-fatty acids, leads to a decrease in ROP development, which can be explained by the antioxidant effect of unsaturated fatty acids that reduces lipid peroxidation [148, 149]. Therefore, it would be a key early intervention strategy to reasonably expect a status with strong antioxidants at birth or provide antioxidant supplementation to reduce the risk of ROP in preterm infants.

## 4. Summary

**Figure 6:**
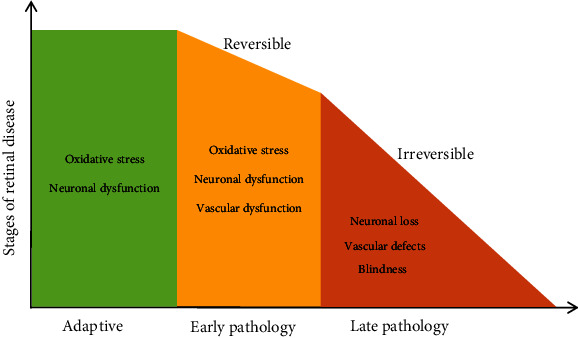
The progression of retinal disease in the hypothetical stages. Effective intervention during the early stage of retinal disease would be most beneficial in protection against vision loss (revised from Machelle T. Pardue, 2018, Prog Retin Eye Res).

An increasing number of people will be diagnosed with retinal diseases in the future. The number of people with vision loss will be 61 million, and that of those with vision impairment will be 470 million by 2050 [3]. The healthcare system will face challenges from millions of people with vision loss and their caregivers. Poor effective treatments currently exist during the advanced disease stage in the clinical setting. Hence, it is important to find ways to detect retinal diseases early and monitor disease progression and treatment efficacy because the adaptive phase and early pathology phase are reversible and are also the most effective phases in retinal diseases (Figure 6) [20].

Oxidative stress plays a critical role in the pathological processes of many types of retinal dysfunctions [150]. Anatomical features of the eye make the retina more susceptible to ROS production, especially with increasing age [151–153]. The retina, especially the photoreceptors and retinal pigment epithelium, is rich in polyunsaturated fatty acids, which are susceptible to lipid peroxidation [50, 154]. Persistent prooxidant factors and decreased antioxidant capacity with age may accelerate oxidative stress. Thus, the increase in ROS and intracellular Ca^2+^ concentrations are common pathological changes in the retina [50]. Many studies have indicated that oxidant stress injury is the first step to induce the cell death of retinal neurons [24, 50, 155–157]. Ocular diseases share the same cellular mechanisms. In the final stages of retinal disease, there are limited effective treatments that may rescue lost vision.

Therefore, early intervention for retinal diseases is necessary and urgent, and may offer hope to improve patients' quality of life through functional vision. First, management of the source of ROS generation is a key factor in preventing the progression of retinal disease. Various factors that lead to ROS generation should be suppressed, including hyperglycemia, intraocular hypertension, hyperlipidemia, ultraviolet light exposure, ischemia, and obesity. Early reduction of these stressors may ameliorate ROS-induced retinal damage. Second, the application of dietary antioxidants, antioxidant supplementation, or pharmacological inhibitors could be an effective intervention strategy in the early stages of retinal diseases. This approach may scavenge excess free radicals and reduce oxidative stress injury. Third, physical exercise as a rehabilitation treatment has shown pluripotent benefits in multiple systems of the body, including retinal diseases [158, 159]. Recently, exercise has been demonstrated to have protective effects in animal models of retinal diseases via multiple mechanisms, including the BDNF/TrkB signaling pathway, increased blood flow, and modulation of VEGF and its receptors [160, 161]. Retrospective studies in humans have also indicated that exercise treatment for visual disorders can improve visual function and quality of life. Therefore, exercise interventions may be implemented in the early stage of retinal disease. The retina is a metabolically active tissue that is susceptible to oxidative injury. Therefore, hepatic injury or hyperlipidemia may induce metabolic turbulence and negatively affect retinal health. Maintaining blood and liver health is beneficial to the retina.

### 4.1. Future Directions

1. Methods for the early detection of retinal dysfunction must be developed to identify the key treatment window and monitor disease progression
2. Simple, accessible self-screening is needed to address the problem of visual function and obtain a clinical examination
3. To explore the differences in the therapeutic effects of nonselective antioxidants, general ROS scavengers, specific inhibitors of Nox isoforms, and molecular/genomic target drugs are necessary to curb the progression of retinal diseases
